# Women suffer more from disrespectful and abusive care than from the labour pain itself: a qualitative study from Women’s perspective

**DOI:** 10.1186/s12884-018-2026-4

**Published:** 2018-10-04

**Authors:** Mengistu Welday Gebremichael, Alemayehu Worku, Araya Abrha Medhanyie, Kerstin Edin, Yemane Berhane

**Affiliations:** 1 College of Health Sciences, Mekelle University, P.O.Box: 1871, Mekelle City, Ethiopia; 2 Addis Continental Institute of Public Health, Addis Ababa City, Ethiopia; 3 School of Public Health, Addis Ababa University, Addis Ababa City, Ethiopia; 4 Sexual and Reproductive Health, the Department of Nursing, Umeå University, Umeå, Sweden

**Keywords:** Respectful maternity care, Disrespect and abuse, Qualitative, Tigray, Ethiopia

## Abstract

**Background:**

Utilization of institutional delivery services could be hampered by women’s experience of disrespectful and abusive care during childbirth. However, such experiences are not well documented and taken into consideration id planning maternal health services in many developing countries. The aim of this study was to describe women’s experience of disrespect and abuse during giving birth at health facilities in northern Ethiopia.

**Methods:**

A qualitative phenomenological study was conducted in Tigray, Ethiopia. Focus group discussions (FGDs) with primipara and multipara women were conducted to collect the necessary information. All study participants had their last delivery at a health facility in the year preceding the study. A semi-structured discussion guide was used to elicit discussion. Discussions were audio recorded and transcribed verbatim in the local language and then translated to English. Data were analyzed using thematic analysis approach assisted by the Open Code qualitative data management software.

**Results:**

The study participants described disrespect and abuse as serious obstacles to utilization of maternal health services. Women reported experiencing feelings of being infantilized, losing self-control, being overlooked, being informed bad news without proper preparation, repeated examination without being properly communicated/informed, disallow companions, and left unattended during labor. Facility related issues include women’s perception of incompetence of professionals attending delivery, unhygienic facilities, and unavailability of basic supplies.

**Conclusion:**

Women consider health facilities not fully prepared to provide respectful maternal care. Sustainable increase in institutional delivery requires ensuring quality, compassionate and caring services in all health facilities.

## Background

Increasing the proportion of deliveries attended by skilled providers is essential to reduce life threatening risks to women and their newborns during child birth. The coverage for institutional delivery has been improving in Ethiopia in recent years reaching 26% in 2016 compared to 10% in 2011 [1]. However, there are growing concerns about the quality of services and about disrespectful and abusive practices during childbirth is widespread [2, 3].

Violations of the principles of compassionate and respectful care during childbirth in maternity facilities has been witnessed widely [4–7], although such experiences could be rampant in low income countries experiences of bullying, coercion, and non-consented procedures have been reported by women in high income countries as well [4, 8]. The severity and frequency of disrespectful and abusive care practices are associated with level of health care setting, qualifications of the care providers, resource availability, service hours, and socio-economic status of women [9].

The care received during pregnancy and childbirth could have an immediate and long-lasting effect on future health care utilization practice [6, 7]. The fear of disrespect and abuse often hold women back from seeking institutional care again [7, 10–12], and that could be a more powerful deterrent to seeking skilled birth care than other more commonly recognized deterrents such as geographic inaccessibility and financial constraints [3]. Women have been observed going to distant health facilities to seek care if they perceive the quality of service to be good [13]. Respectful and compassionate care is considered as women’s basic human right [14]. Violation of respectful care practices during childbirth has been recently recognized as a priority issue by the World Health Organization in improving maternal health care utilization coverage and quality [15] and many governments and implementing organizations have taken various corrective and punitive actions [16, 17].

Ethiopia has incorporated compassionate and respectful maternity care in its latest health sector strategic plan [18]. However, the extent and types of disrespect and abuse in the Ethiopian context are not well documented as in many other low income countries [3]. It is imperative to have contextual understanding of disrespectful and abusive care as experiences are likely to differ by context [3, 14]. This study was thus conducted to understand experiences of women who utilized institutional delivery services during their most recent birth in Northern Ethiopia.

## Methods

### Study area

The study was conducted in Tigray National Regional State, northern part of Ethiopia where the reported antenatal care coverage was 100%, institutional delivery was 55.3%, and postnatal care utilization rate was 69.6% [19]. Context specific strategies were used in the region to overcome reasons that favor home delivery. For instance, there is a belief that if a woman fails to have a hot drink or food immediately after birth, she could be exposed to evil spirits that can harm her health. The region mobilized volunteers in collaboration with local women development groups to prepare and serve hot porridge to women immediately after childbirth in the health institutions. In addition, strengthening ambulance services and establishing maternity waiting homes has shown good results [20].

### Study methods

We used a qualitative phenomenological research approach. Women who gave birth in health institutions within a year preceding the study were eligible. Study participants were selected through a purposive sampling procedure that considered their residence (rural and urban), the health facility type (health center or hospital) and their parity (primipara or multipara). A total of eight FGDs were conducted, four at health centers and four at hospitals. The recruitment of study participants was facilitated by health extension workers (community health agents) in the study areas.

### Data collection procedure

Each focus group discussion (FGD) involved 7–8 women and discussions were held in locations outside health facilities that were agreed by the participants. U-shaped seating arrangements were used during the discussions so that participants can face each other and the moderator could also observe everyone. An FGD took on average 75 to 90 min. Information about the purpose of the study and procedures during the discussion were explained to obtain informed consent from each participant. FGDs were facilitated using discussion guide that contained discussion points addressing issues related to quality of maternity and delivery services, barriers to utilizing health institutions for delivery, interaction with health professionals, and disrespectful and abusive practices in maternity care. The FGDs were facilitated by two people; the moderator and the note taker. All FGDs were conducted in the local language (Tigrigna) and audio recorded using a digital recorder.

### Data analysis

The recorded materials were transcribed verbatim in the local language *Tigrigna* and then translated into English by research assistants. Each FGD was thoroughly read and re-read to develop a coding scheme by the first author and reached consensus on the coding approach with the other authors. Coding was completed by going line by line through the material assisted by a qualitative data management software, called open code, which was developed by Umea University in Sweden and freely available to users [21]. Codes were organized to create categories and themes. Analysis was done using thematic analysis approach.

## Results

A total of 62 women who gave birth in the year prior to this study participated in eight FGDs. Four FGDs were held with urban dwellers and the remaining four groups were with rural dwellers. In both settings, two of the four FGDs were with primipara women and the other two with women who had more than one birth experience.

The findings of the study are summarized in three major thematic areas: losing control of the process, reactions of care recipients to the care received during labour and delivery, and resource scarcity as contributor to disrespectful and abusive care (Table 1).

**Table 1:** Construction of study categories and study themes

Codes	Categories	Themes
- Repeated examination and examination without finding notification - Ignoring or undermining pain - Being told bad news - Inconsistent information - Shouting and insulting - Restraining for long	- Being infantilized - Undermine woman’s feeling and experiences	Losing control of the childbirth process
- Shifts bring difference - Disallowed to have companionship - Admitted against consensus - No assistance near by	- Obliged to do self-care - Care against will - Privacy as time dependent - Humilating/degrading	
- Declined to be examined - Care provider preference - Noticed institution difference - Short stay at health facility	- Not willing to use the service again - Projecting the outcome - Fear of being hurt related to negligence and incompetence - Felt ok for stayed short in institution	Reactions of care recipients to the care received during labour and delivery
- Unhygienic latrine - Staff work burden	- Scarcity of running water - Staff shortage	Resource scarcity as contributor to disrespectful and abusive care

### Losing control of the childbirth process

The women recognized the differences in professionals’ approach as well as their preference. Women were positive about professionals who were supportive, friendly, polite, and who stayed close for their needs. Women even preferred professionals who did nothing but spoke to them with encouraging words and showed sympathy. On the contrary, women expressed hatred for those who were cruel, not compassionate and showed unfriendly facial expressions. Women mentioned disrespect and abuse by providers that ranged from ignored needs to pain inflicted during procedures.

Although the women had no complaints about abdominal and vaginal examinations they reported disappointment because they were not told the findings of the examinations. In health facilities were training happens, providers including trainees perform physical examinations turn by turn without telling the women their impressions and findings, which annoyed women.

A woman from the urban area said, *“I was examined and told my labour is at early stage … at that point my baby was on the way out but I was restricted to stay in my left side …I told my care provider I am urged to push down and requested for help …he said I just examined you (you are not yet ready) and ignored me and continued playing with his mobile phone ...the urge to push down was irresistible, I then turned on my back by myself and gave birth…then the care provider tried to assist, my child was not crying immediately … I think it was suffocated for long as the provider ignored my call (and delayed the process)…luckily my baby survived but I was not happy with the care.”*Another woman who had twins in her first birth by caesarean section said*, “I was threatened by the care providers when I could not obey their order to move around in the post-operative period because I had severe pain post cesarean section. The provider shouted at me… get up … with no support and while in pain”.*

Providers sometimes force women to stay at health institution to prevent home delivery. Women visiting health facilities for routine antenatal care follow up are forced to stay at health institution without their consent.

A woman from the urban area said, *“I was kept at the health institution without having labour pain or any indication it may start soon…they did several examinations and I was sent home after one-day admission because my labour did not start. One month later, I was again admitted to the facility because the presentation of my baby was not normal (it was breach presentation)…I believe the position changed from normal to abnormal due to the repeated abdominal examinations they did previously”.*

Women reported some providers were incompetent and negligent. Women also reported observing junior care providers being guided by senior care providers to perform certain procedure…junior providers take longer time to perform tasks and often left unsupervised by senior care providers. Women admitted to hospital also complained lack of support for basic physiologic needs during long labor hours because family members are not allowed to accompany them, which left some women to stay in uncomfortable conditions soaked with blood and urine.

A woman said, “*she had bleeding, no one was around... bleeding became profuse …when the provider reached and examined her, she was weak and her blood pressure was very low. I was treated with three bags of intravenous fluid, which was immediately followed by blood transfusion. Providers ignored me and were chatting a lot among themselves… and fail to react timely in case of emergency.*”

Women has no control over their own situation and often felt infantilized. The response of health providers to women’s agony in labour pain was unsympathetic and sometimes overlooked.. Women in labour also suffer from lack of privacy. Having a group of providers standing round a labouring woman is terrifying for her; even worse is when providers seat on the foot of the bed. Providers’ behavior became worse at night shifts while they were also few in number:

A woman said, “*I was asked to change bed without being properly cleaned and my blood still dripping on the floor…the providers behavior get worse during night shift … I will not give birth again in health institutions”.*

Women are often left to worry about the outcome of their pregnancy and often with fear of having a bad outcome. Women are highly sensitive and could be easily disturbed with any negative words from providers, intentional or unintentional, about the progress of either their labour or the status of the baby. Bad news cause stress and discomfort to women in labour. Provider’s approach to disclose bad news such as the absence of the baby’s heartbeat was considered rude.

### Reactions of care recipients to the care during labour and delivery

Experiences during childbirth could leave lasting positive and negative impressions in the minds of the mother. Some leave with good impression and some other regretted having birth in a health facility due to undignified care they received in the facilities.

Participants from the rural area said, *“health providers were caring during labour and delivery …it was similar to the care you can get at home”…* “*These days, St. Mary was with them (to the providers, they were so good)… everything has been smooth”.*

Another woman from a rural area said,*“(This woman initially said the care she received during labour and delivery was good …but later in the discussion, after hesitating a bit), she said, she was restricted to lie on one side and when she felt discomfort and tried to change position…, the response from the care provider was bad..., the provider threaten to collide me to the wall...I hate them(providers), they made my labour and delivery process stressful. I really hate them; they put me in stress the whole day…the provider later simply brought a pair of scissor, which was terrifying, and cut me...*I thought of never going to the health facility again … it is better to give birth at home with dignity…(after a little pause)…*however, after everything went well, I changed my mind…had it been at home, giving birth to such big baby could have been disastrous…I could have lost my life(showing a sad face).*

A woman from the urban group who gave birth to her first child said, “*The care providers hurt us psychologically. They came and did vaginal examinations repeatedly as simple as anything but it is a big trauma to us*”,(expressed her emotion by increasing her voice and showing anger on her face)…*I really felt pain after the examination…I tried to repeated vaginal examinations…only to receive an insult…“shut up” you conceived for your own and now you joke on us…they were not also well organized and often did not communicate well to one another…so the same examination is done repeatedly unnecessarily”*

### Resource scarcity as contributor to disrespectful and abusive care

Clients in rural health centers tried to justify the behaviors of health providers. Clients recognized health providers are few in number, most were junior, and they serve long duty hours. Clients also recognize scarcity of resources in health facilities as hindrances to rendering proper and respectful maternity care services. In some health facilities, water is not available at all and sanitary facilities are unhygienic. Shortage of consumable materials such as suturing needles could keep women waiting on the delivery couch for hours.

A woman who was giving birth her first child said,*” I got my episiotomy sutured after hours of waiting because they had no needles ready to suture.”*

A woman from an urban area who delivered her first child said, *“I was left alone on a couch for 7 hours with instruction to remain in the same position, which was inconvenient and very cold without no beddings. Then after …they forced me stand up and move…I was not strong enough yet. What annoyed me most was they made me collect my blood stained linens and clothes.*

A multipara woman from the urban group said*, “I gave birth in the middle of the night (around 3 A.M.) and was left on the same couch on which I delivered until 8 A.M.in the morning…there was no bed to transfer me on after delivery. I spent hours in uncomfortable position and I got back pain.”*

## Discussion

This study illustrates the range of experiences women endured during labour and delivery in health facilities. Disrespect and abuse left women with lasting negative feelings and fear of experiencing the same in case they need to use the facilities again, Baas described the same feelings [22].

We found disrespectful and abusive experiences were more common during night shifts in agreement with previous reports [9]. Previous reports also indicated night shifts are associated with greater verbal and physical abuse [9], and women are more likely to be left alone during night shifts [23]. On the other hand women who delivered during day time complained about having too many trainees around them during day time and more violation of privacy [24].

Abandonment and neglect were common, mainly reported by rural participants [9, 25]. Women left an attended or not having a relative to support them could endanger the lives of the mother and the newborn. Deprivation of companionship during labour was reported repeatedly by urban participants and elsewhere [26]. Companions during labour can provide physical and psychological care and facilities need to consider allowing companions especially when adequate number of care providers are not available [4].

Poor provider-client interactions, lack of privacy, discrimination against cultural practices, physical abuse, dirty facilities, and delays in receiving care reported by this study have also been consistently reported in many other contexts as reasons for dissatisfaction with health facility maternity services or for not giving birth at facilities [2, 7, 27–30]. Provision of proper training and orientation for health care providers on the importance of maintaining respectful and compassionate care at all time is critical to achieving universal skilled delivery coverage [31]. Providers with sympathy and compassion can attract more clients regardless of their level of competence [24]. Shifting efforts to establishing strong and continuous support mechanisms for health institutions and providers could be a better approach instead of trying to force women to give birth in ill prepared health facilities using various sanctions such threatening to withhold social visits and involvement in some religious ceremonies; as has been attempted in the study areas.

The strength of this study can be ascribed to the research methods. The study, unlike many previous studies that were health facility-based, was a community-based and women were able to talk about the experiences more freely. The quality of the transcription was re-checked by transcribing part of the material again and comparing with the original transcription. Although a qualitative study by its nature is not supposed to allow generalizability, by including both rural and urban clients served at various levels of health care in the study we strongly believe the findings fairly reflect the general situation in many parts of the country as the health service organization, the curriculum being used for training health workers, and the supportive supervision schemes are more or less the same throughout the country.

## Conclusions

The disrespectful and abusive care experienced appears to be rampant in health facilities and women in labour are enduring unacceptable pain and sufferings. That is a real hindrance to achieving universal coverage for skilled delivery. Strategies for respectful and compassionate care during every delivery need to be an integral part of any effort to increase institutional delivery.
